# AUXIN RESPONSE FACTORs: from transcription factors to auxin effectors

**DOI:** 10.1111/nph.71477

**Published:** 2026-08-13

**Authors:** Melissa Dipp‐Álvarez, Jorge Hernández‐García

**Affiliations:** ^1^ Laboratory of Biochemistry Wageningen University Stippeneng 4 Wageningen 6708WE the Netherlands; ^2^ Centro de Biotecnología y Genómica de Plantas Universidad Politécnica de Madrid (UPM) – Instituto Nacional de Investigación y Tecnología Agraria y Alimentaria (INIA/CSIC), Campus de Montegancedo UPM Pozuelo de Alarcón 28223 Spain

**Keywords:** auxin, molecular evolution, plant evolution, signalling pathway, transcription factor

## Abstract

Signalling pathways have originated and diversified extensively during evolution. Using AUXIN RESPONSE FACTORs (ARFs) as a model, we examine how a plant signalling system evolved through the co‐option of pre‐existing transcriptional regulators, a principle well established in evolutionary biology in organisms, such as animals. ARFs are an ancient family of DNA‐binding proteins that predate the canonical nuclear auxin pathway and likely acted as auxin‐independent transcriptional repressors. The emergence of auxin signalling involved coupling this ancestral transcriptional module to a regulatory system based on a series of co‐repressors regulated through F‐box‐mediated degradation, thereby conferring signal responsiveness without altering core ARF biochemical properties. Molecular diversification and innovation – through gene duplication, acquisition of transcriptional activation, and changes in interaction partners – expanded the developmental outputs controlled by auxin while preserving the underlying regulatory logic. Beyond their role as auxin effectors, ARFs function as integrative hubs that coordinate multiple developmental and environmental inputs. Using this family as an example, we illustrate how signalling pathways are built by rewiring existing regulatory frameworks, providing general principles for understanding the evolution and function of plant signalling networks.

## Graphical abstract

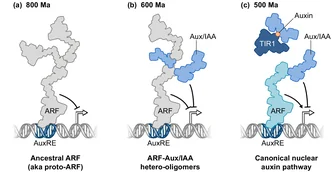

**Table:** 

Content		
	Summary	607
I.	Introduction	607
II.	Before signalling: stripping the A from ARFs	608
III.	Becoming signal‐responsive: from A to ARF	608
IV.	Diversification and developmental specificity: from ARF to ABC	610
V.	Beyond auxin: ARFs as integrators	611
VI.	General lessons: signalling pathways built around TFs	611
	Acknowledgements	612
	References	612

## I. Introduction

Signalling pathways are often described as linear connections from a signal to a response. In most cases, this response is attributable to changes in gene expression (i.e. transcription). However, this idea raises a fundamental evolutionary question: how do such connections form? Transcription factors (TFs) and transcriptional regulators (TRs) are the common signalling pathway endpoints leading to transcriptional changes. This is true for classical plant hormone signalling pathways, such as auxin, gibberellins, or strigolactones (Blázquez *et al*., 2020). The origin of these signalling pathways required different elements, for example signal perception, regulatory proteins, and transcriptional effectors, to first arise and then interconnect. Accumulating evidence suggests that TFs/TRs predate the signalling pathways in which they operate (Peñuelas *et al*., 2019; Ferreira‐Guerra *et al*., 2020; Hernández‐García *et al*., 2021; Carrillo‐Carrasco *et al*., 2023). Signalling systems frequently emerge by coupling pre‐existing elements to new regulatory modules. Understanding how this coupling occurs is key not only to explaining how signalling pathways evolve, but also to understand the molecular basis of their function.

**Fig. 1:**
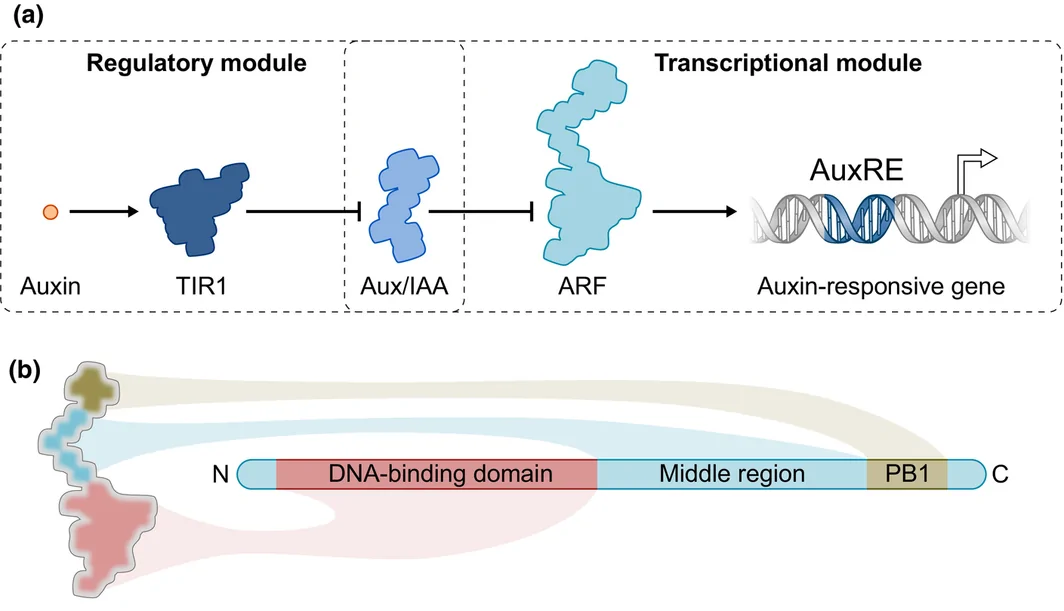
AUXIN RESPONSE FACTORs (ARFs) and the nuclear auxin pathway. (a) The nuclear auxin signalling pathway cascade showing the main elements. Division into a regulatory sensing module and a transcriptional module is highlighted. (b) Cartoon (left) showing the different structural and functional ARF protein domains and equivalence with a linear representation of ARF architecture (right) from the amino‐terminal (N) to the carboxy‐terminal (C). PB1, Phox and Bem1. Linear representation is approximately scaled to the ARF domains mean size. TIR1, TRANSPORT INHIBITOR RESPONSE1; Aux/IAA, AUXIN/INDOLE‐3‐ACETIC ACID; AuxRE, auxin response element.

The AUXIN RESPONSE FACTOR (ARF) family of TFs provides a tractable example of this coupling. Best known for their role in auxin‐mediated transcription in land plants, ARFs are a billion‐year‐old family of DNA‐binding transcriptional effectors that functioned before the emergence of the nuclear auxin signalling pathway (NAP; Fig. 1a). Here, we use ARFs as a case study to argue that signalling pathways arise by the evolutionary co‐option of pre‐existing regulators, and subsequent diversification operates within this inherited regulatory framework.

## II. Before signalling: stripping the A from ARFs

ARFs have been associated with auxin since their discovery in the late 1990s (Chapman & Estelle, 2009). Yet, this does not reflect their evolutionary history. ARFs are found in all streptophytes, suggesting an early origin > 800 million years ago (Ma; Mutte *et al*., 2018). However, algal ARFs are not connected to a canonical auxin response pathway: key components, including the AUXIN/INDOLE‐3‐ACETIC ACID (Aux/IAA)‐mediated repression coupled to TRANSPORT INHIBITOR RESPONSE1 (TIR1)/AUXIN SIGNALING F‐BOX (AFB)‐dependent degradation, are absent or lack essential motifs. While this could reflect the loss of an ancestral NAP‐regulated ARF, the paraphyly of streptophytes supports a more parsimonious scenario: the lack of connection to auxin responses represents the ancestral state of ARFs (Carrillo‐Carrasco *et al*., 2023).

ARF domain architecture is conserved among all lineages (Fig. 1b; Hernández‐García *et al*., 2024): an amino‐terminal DNA‐binding domain (DBD), a central middle region (MR), and a carboxy‐terminal Phox‐Bem1 (PB1) domain. The DBD contains a B3 domain that confers specific binding to auxin response *cis* elements (AuxRE). While AuxRE were discovered in the context of auxin response (Ulmasov *et al*., 1995), these DNA elements exist in algae, and algal ARFs specifically recognize these elements (Martin‐Arevalillo *et al*., 2019; Hernández‐García *et al*., 2024). This suggests that AuxRE and ARFs formed a *cis*–*trans* regulatory duo before the emergence of the NAP. The MR, a large intrinsically disordered region, is involved in transcriptional regulation. Available evidence suggests that most extant ARFs act as transcriptional repressors, consistent with the prediction of an ancestral repressive function (Hernández‐García *et al*., 2024). Thus, core aspects of ARF function predate auxin responsiveness. Consistently, transcriptional responses to auxin in streptophyte algae overlap with other responses and are not enriched for AuxREs, supporting the lack of ARF‐mediated auxin response (Carrillo‐Carrasco *et al*., 2025).

The PB1 domain mediates head‐to‐tail protein–protein interactions and enables oligomerization (Mutte & Weijers, 2020). This is an ancestral property possibly enabling the formation of higher‐order regulatory complexes. Importantly, PB1s provided a pre‐existing interaction interface underlying auxin‐dependent regulation as they mediate the interaction between Aux/IAAs and ARFs (Korasick *et al*., 2014; Nanao *et al*., 2014).

**Fig. 2:**
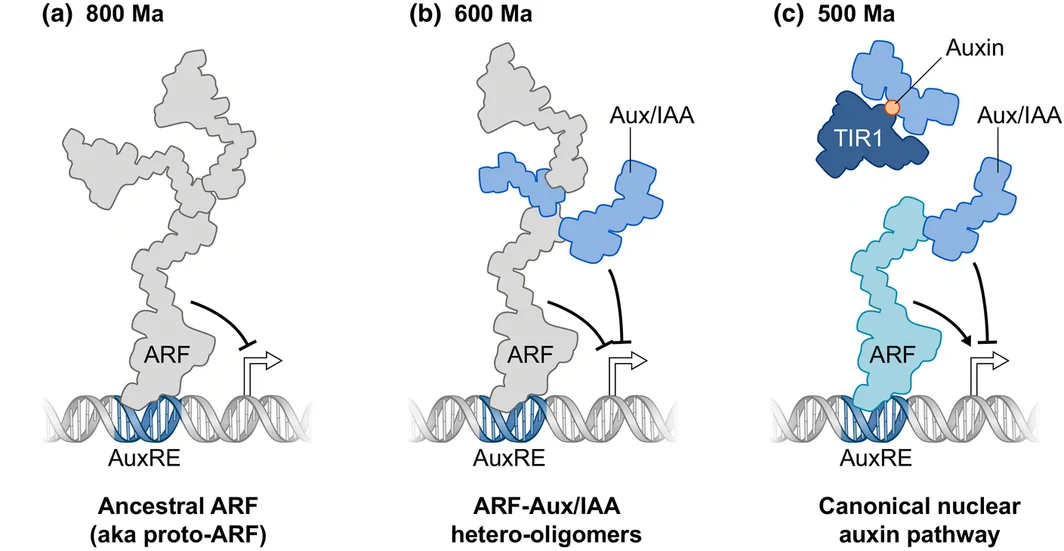
Molecular evolution of AUXIN RESPONSE FACTOR (ARF) effector function. Evolutionary history of the inferred ARF molecular effect on gene expression with predicted dates. (a) Ancestrally predicted function, including auxin response element (AuxRE) binding, PB1‐driven oligomerization and transcriptional repression. (b) Next step in evolution was the appearance of AUXIN/INDOLE‐3‐ACETIC ACID (Aux/IAA), able to act as co‐repressors together with ARFs through oligomerization. (c) The last step in effector function is the acquisition of transcriptional activation, and the appearance of Aux/IAA destabilization through the TRANSPORT INHIBITOR RESPONSE1 (TIR1)–auxin, that is, the evolution of the nuclear auxin signalling pathway (NAP). Ma, million years ago.

This view suggests that ARFs functioned as TRs before becoming auxin‐responsive (Fig. 2a). In fact, some extant ARFs, such as those in algae, likely function in an auxin‐agnostic manner (Hernández‐García *et al*., 2024).

## III. Becoming signal‐responsive: from A to ARF

The transition from auxin‐independent to auxin‐responsive ARFs is a key event for the origin of the NAP. In the pathway (Fig. 1a), Aux/IAA proteins interact with ARFs and repress transcription. When auxin accumulates, the TIR1/AFB F‐box receptors promote Aux/IAA proteasomal degradation, enabling transcriptional activation by ARFs (Tiwari *et al*., 2001; Dharmasiri *et al*., 2005; Kepinski & Leyser, 2005). This mechanism does not alter the intrinsic DNA‐binding capacity or effector function of ARFs, but utilizes Aux/IAA ability to repress transcription to act as the signal‐dependent switch. This effectively allows the regulatory module – receptor and repressor – to regulate the transcriptional module – ARF and AuxREs. While the overall logic of the NAP is well understood, the evolutionary sequence by which its components assembled remains unclear.

Signalling pathways likely emerge following different evolutionary roads. In some, a pre‐existing perception module may later become connected to transcriptional responses; in others, pre‐existing TFs may acquire responsiveness through newly evolved perception regulatory modules. Instances of the former have not been described in plants, although they are hypothesized for the evolution of KAI2/D14 signalling (Wang *et al*., 2022). Conversely, the co‐option of pre‐existing transcriptional modules seems to lie behind the origin of most known signalling pathways (Hernández‐García *et al*., 2019, 2024; Peñuelas *et al*., 2019; Ferreira‐Guerra *et al*., 2020). While the specific molecular events leading to evolutionary co‐option differ, the exploitation of a pre‐existing TR remains a common principle in plant signalling pathway evolution.

The appearance of signal‐regulated proteolysis systems stands out: this is the case for gibberellins, strigolactones, jasmonates, or auxins (Blázquez *et al*., 2020). The NAP system utilizes auxin as a glue to bring together the TIR1/AFB and the Aux/IAAs (Tan *et al*., 2007). This mechanism matches that of jasmonates, which allows the CORONATINE INSENSITIVE1 (COI1) F‐box to interact and destabilize the JAZ repressors (Chini *et al*., 2007; Thines *et al*., 2007). In fact, TIR1 and COI1 encoding genes arose from gene duplication in a common land plant ancestor (Mutte *et al*., 2018). This indicates both F‐boxes acquired the ability to bind two different signalling molecules to regulate two different transcriptional modules.

An additional evolutionary intermediate stage likely facilitated the evolution of the NAP: Aux/IAA appeared *c.* 600 Ma after the divergence of the Klebsormidiophyceae and the branch leading to the crown streptophytes (Mutte *et al*., 2018). While inconclusive, structural and phylogenetic data suggest that the Aux/IAAs appeared from a partial duplicate of an ARF gene (Hernández‐García *et al*., 2024). As algal Aux/IAA PB1 can interact with those of algal ARFs (Hernández‐García *et al*., 2024), it is tempting to speculate that this ancestral Aux/IAA, retaining ARF repressor function and the oligomerization ability provided by the PB1, probably acted as a constitutive ARF co‐repressor (Fig. 2b). In any case, it was only in a land plant common ancestor that Aux/IAA acquired the degron motif recognized by the TIR1‐auxin complex (Mutte *et al*., 2018).

The success of the NAP functionality also required the evolution of ARF transcriptional activation (Hernández‐García *et al*., 2024). Without this ability, Aux/IAA and ARF theoretically perform an identical transcriptional output: repression. These would likely leave auxin‐dependent degradation of Aux/IAA as a weak trigger devoid of the transcriptional response range auxin has in land plants (Kato *et al*., 2020). While a repressor–repressor transcriptional module was likely the ancestral chassis upon which a repressor–activator module later evolved (Fig. 2b,c), it remains unresolved whether the perception module was coupled to an already diversified ARF system or to a more ancestral, uniformly repressive one. In fact, the evolution of transcriptional activation in ARFs and the appearance of the perception module could have occurred in a very narrow evolutionary period in the land plant ancestor's branch. It is, nevertheless, plausible that intermediate scenarios occurred. For example, it is possible that additional co‐activators competed with Aux/IAA to physically interact with repressor ARFs, allowing for a robust and dynamic NAP system to function without intrinsic ARF activation capabilities. Even with the lack of a NAP as we know today, controlling co‐repressor stability could still render relevant transcriptional implications, for example if the repressive activity of ARF was significantly weaker than the one combined with Aux/IAA.

Evolution of an alternative NAP seems to have occurred more recently without the requirement of an independent regulatory and perception module. Reminiscent of bacterial TFs and some of the metazoan hormonal receptors that act as both sensors and effectors (Baker *et al*., 2013; Conway *et al*., 2022), some ARFs show sensing capabilities that bypass the canonical NAP. This is shown in the case of ETTIN/ARF3 (Kuhn *et al*., 2020), which perceives auxin, disrupting the co‐repressor interaction, thus allowing for differential gene regulation directly. This is an extreme case of pathway reduction specific to the ARF3 clade that evolved in angiosperms (Ang *et al*., 2024), with no other reports of signalling pathway effectors becoming a direct sensor of the same signal.

Together, these scenarios converge on a central idea: auxin signalling did not arise by inventing new transcriptional effectors but by progressively wiring regulatory control onto an existing ARF‐based transcriptional system.

## IV. Diversification and developmental specificity: from ARF to ABC

**Fig. 3:**
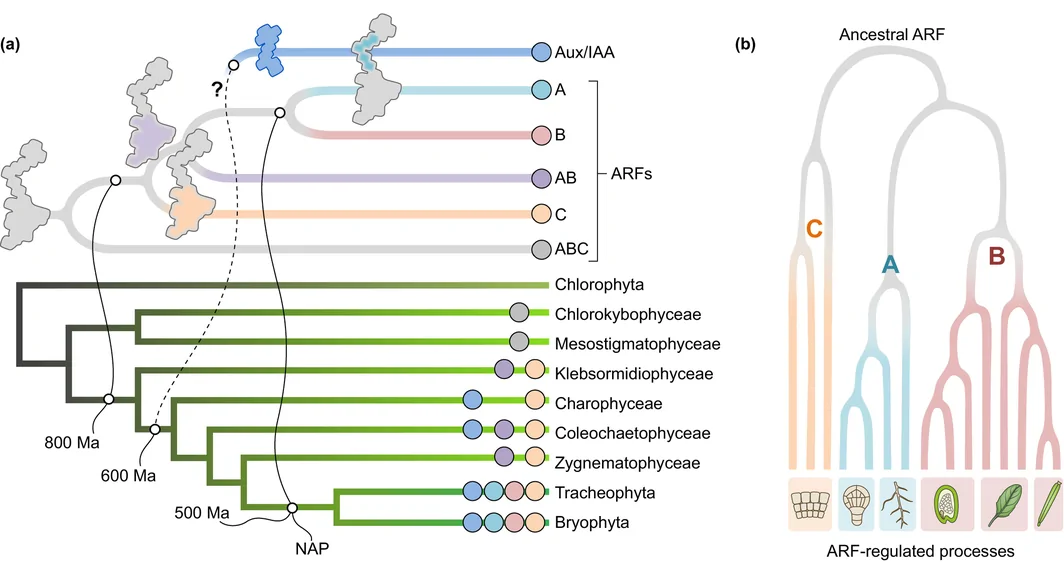
Expansion and functional divergence of the AUXIN RESPONSE FACTOR (ARF) family. (a) Major molecular innovation and divergence events in ARFs during streptophyte evolution, pinned to both the ARF family (upper) and lineage (species) tree (lower). Gene and species tree based on Mutte *et al*. (2018) and Hernández‐García *et al*. (2024). Ma, million years ago. (b) Simplified tree of ARF family and class‐specific expansions in land plants linked to developmental specialization. Icons represent different ARF‐regulated processes in angiosperms; from left to right: cell proliferation and differentiation, embryo development, root development, seed development/endosperm cellularization, leaf development, and gynoecium development. NAP, nuclear auxin pathway. Note that in (a), NAP is pinpointed to an ancestor of all land plants where it is predicted to have originated.

Once ARFs were incorporated into the NAP, their pre‐existing modular architecture provided a substrate for diversification, together with an expansion of the developmental outputs controlled by auxin. Before this, phylogenetic reconstructions indicate that the major branching events giving rise to the A/B/C‐classes predated land plant diversification and arose through local gene duplications rather than through whole‐genome duplication events, followed by functional divergence (Mutte *et al*., 2018; Flores‐Sandoval *et al*., 2018; Hernández‐García *et al*., 2024; Fig. 3a). The earliest split, separating AB‐ and C‐class ARFs, occurred in a common ancestor of Klebsormidiophyceae and Phragmoplastophyta and is associated with DBD specialization (Hernández‐García *et al*., 2024). A second gene duplication event occurred in an AB‐ARF of a land plant common ancestor, giving rise to the A‐ and B‐class lineages. This step is associated with the emergence of a discrete region within the MR, endowing activation capacity to the A‐class through the interaction with eukaryotic co‐activation complexes (Hernández‐García *et al*., 2024, 2026), an important step towards the NAP acquiring its full transcriptional capabilities. A‐ and B‐classes inherited the DNA‐binding mode and degradation via proteolytic control from an AB‐ARF common ancestor (de Roij *et al*., 2025; Rienstra *et al*., 2025), which could imply that hormone‐responsive control was acquired onto an already diversified A and B‐ARF scaffold.

Following the emergence of A‐, B‐, and C‐class ARFs, whole‐genome duplications, and lineage‐specific subfunctionalization (Mutte *et al*., 2018) in land plants, especially in euphyllophytes, produced a wide array of ARFs specializing in a variety of developmental outputs (Fig. 3b). Importantly, these later events did not alter the basic logic of NAP signalling, but instead produced distinct transcriptional outputs by reusing the same regulatory framework. As drivers of auxin‐dependent transcriptional responses, A‐class ARFs illustrate how gene expansions can lead to developmental specialization without changing the core logic of a signalling pathway (Gera *et al*., 2022). In *Arabidopsis*, five A‐class paralogs control distinct root developmental programmes, with AtARF5 contributing to primary root formation, AtARF7/19 to lateral root development, and AtARF6/8 to adventitious rooting (Okushima *et al*., 2005, 2007; Gutierrez *et al*., 2009; Schlereth *et al*., 2010; Zhang *et al*., 2023). Across these contexts, the same auxin response framework is reused, but with different developmental roles and distinct ARF‐partner combinations. Little to no differences exist in DNA *cis*‐element recognition between A‐class ARFs (Galli *et al*., 2018; Freire‐Rios *et al*., 2020; Rienstra *et al*., 2025), which seem to have diverged through changes in expression domains, interaction partners, and, altogether, in their integration with other regulatory pathways. Similarly, B‐class ARFs DNA binding has undergone little divergence, even compared with A‐class ARFs (Boer *et al*., 2014; Galli *et al*., 2018). Still, specific neofunctionalization events have occurred outside the DBD to control specialized processes as in the case of the already mentioned ETTIN/ARF3 clade in gynoecium patterning (McLaughlin *et al*., 2025). Interestingly, B‐class has expanded independently within different lineages (Mutte *et al*., 2018). For example, Arabidopsis contains 15 B‐class ARF encoding genes, most of which originate from a Brassicaceae‐specific centromeric B‐ARF cluster involved in endosperm cellularization (Butel *et al*., 2024). However, these expansions do not seem to have produced major biochemical changes in the B‐class, which retain their function to prevent auxin surges by competing with A‐class ARFs (Butel *et al*., 2024).

Contrasting A‐ and B‐class, the C‐class ARFs are the more understudied of the family, yet a common theme emerges across embryophytes: together with *miR160* regulation, they seem to modulate the balance between proliferation and differentiation (Flores‐Sandoval *et al*., 2018; Dai *et al*., 2021). In addition, C‐class biochemical function seems completely conserved from algal C‐ARFs to embryophytan ones, suggesting very little molecular divergence (Hernández‐García *et al*., 2024).

Functional diversification across ARF classes appears to rest on four recurring evolutionary mechanisms: first, changes in DNA‐binding properties (evident in the AB/C split), retaining the core TGTCnn recognition but with minor alterations of the binding‐site preferences and spacing tolerance (Rienstra *et al*., 2025). Second, the evolution of transcriptional activation in the A‐class introduced a major functional asymmetry into a mainly repressive system. Third, context‐dependent interactions diversified ARF outputs through recruitment of distinct partners, including WOX factors in organogenesis (Zhang *et al*., 2023) and TPL‐associated repressors like AIP1/2 and SAP18 (Cheng *et al*., 2026). Finally, paralog‐specific expression domains positioned these conserved regulatory modules in different developmental contexts. Together, these mechanisms generated a family of TFs with distinct developmental roles while preserving the overall logic of auxin‐responsive transcription.

Notably, diversification may also predate the full establishment of A‐, B‐, and C‐class functions in land plants. Streptophyte algal genomes contain multiple AB‐class ARFs (Jiao *et al*., 2020; Feng *et al*., 2024), suggesting that ARF diversification has also occurred in algae, although the functional consequences of this expansion remain largely unknown.

## V. Beyond auxin: ARFs as integrators

Although ARFs are typically introduced as the transcriptional effectors of auxin response, this view is too narrow. Once TFs become embedded in signalling pathways, they often act less as endpoints and more as integration nodes, with their final effect on gene expression shaped by the chromatin environment, the presence of other TFs and co‐effectors, and the regulation these other proteins may be subjected to (Spitz & Furlong, 2012). A classic example is the molecular module involved in hypocotyl elongation in Arabidopsis, where auxin responses are integrated with brassinosteroids, gibberellins, light, temperature, and circadian inputs (Oh *et al*., 2014). In this context, ARF6 cooperates with BZR1 and PIF4 at common target promoters, showing how ARFs connect auxin signalling with broader transcriptional circuits.

This integrative role is also evident in what is known about how ARFs select their targets. ARF binding depends not only on the presence and accessibility of AuxREs but also on motif configuration and cofactor availability. Recent findings indicate that some changes in target specificity occur outside the TF DNA‐binding domain (Gera *et al*., 2022; Rienstra *et al*., 2025), helping to explain how conserved ARFs can still acquire new regulatory behaviours without losing their ancestral binding capacity. Chromatin provides an additional layer of control, influencing both target accessibility and the activity of recruited co‐regulators (O'Malley *et al*., 2016). Post‐translational regulation further modulates ARF accumulation, localization, stability, and activity (de Roij *et al*., 2024). Together, these observations indicate that ARFs are not simply auxin‐responsive TFs, but components of larger regulatory networks that integrate hormonal, developmental, and environmental information.

## VI. General lessons: signalling pathways built around TFs

ARFs provide a useful model to understand how signalling pathways evolve around pre‐existing TRs. This history highlights three recurring features of pathway evolution. First, signalling pathways can arise by imposing conditional regulation onto pre‐existing transcriptional modules. ARFs did not originate as auxin‐responsive effectors; rather, auxin signalling evolved around them by coupling their activity to Aux/IAA‐mediated repression and Aux/IAA to signal‐dependent degradation. Second, the core regulatory logic of the system can remain remarkably conserved, while diversity emerges through context. Despite extensive gene duplication and functional specialization, the fundamental architecture of ARF‐mediated transcription has remained largely unchanged. Third, functional diversification often relies more on changes in regulatory connectivity than on DNA‐binding specificity. ARFs illustrate how deeply conserved DNA‐binding preferences can coexist with broad functional diversification driven by protein–protein interactions and regulatory environment. These principles suggest that the evolution of signalling pathways relies more on rewiring existing ones than on inventing new mechanisms. As such, ARFs are not merely components of auxin signalling; they provided the scaffold around which the auxin pathway evolved.

Yet, several key questions remain open. The ancestral functions of ARFs before the emergence of the NAP are still poorly understood. It is also unclear in which order the core features of the modern pathway were assembled, including Aux/IAA emergence, degron acquisition, and the evolution of transcriptional activation in the A‐class. Algal ARFs have not been functionally characterized in their native context; their comparative analyses could potentially resolve some of the unknowns on how the A/B‐classes have diversified.

## Competing interests

None declared.

## Disclaimer

The New Phytologist Foundation remains neutral with regard to jurisdictional claims in maps and in any institutional affiliations.
